# Risk Factors for Adverse Outcomes in Cancer Patients With Sepsis

**DOI:** 10.14740/jocmr6455

**Published:** 2026-02-28

**Authors:** Christopher Pope, Priscilla Ahwin, Nikhil Kota, Ann Palathingal, Jason Peng, Harshini Suresh, Subhadra Thampi, Krystal Hunter, Satyajeet Roy

**Affiliations:** a Department of Medicine, Cooper University Health Care, Camden, NJ, USA; b Cooper Medical School of Rowan University, Camden, NJ, USA

**Keywords:** Sepsis, Cancer and sepsis, Cancer outcome, Cancer length of stay, Sepsis mortality

## Abstract

**Background:**

Cancer and its various treatment modalities increase susceptibility to the development of sepsis. Because of the complex relationship between sepsis and cancer, we aimed to study the differences in risk factors and outcomes of sepsis in patients with cancer (SCa) compared to patients without cancer (SnoCa).

**Methods:**

A retrospective cohort analysis of all adult patients who received care for sepsis in an urban tertiary healthcare center was conducted. Risk factors and outcomes were compared between the SCa and SnoCa groups.

**Results:**

SCa group (n = 310) was older than SnoCa group (n = 628) (66.8 vs. 61.5 years; P < 0.01). There were higher associations of certain variables in the SCa group compared to the SnoCa group, such as male sex (55.8% vs. 48.2%; P = 0.03), White race (60.6% vs. 51.7%; P = 0.01), lower body mass index (BMI) (28.10 ± 9.3 vs. 30.02 ± 10.4 kg/m^2^; P = 0.01), and history of transient ischemic attack (TIA) (6.1% vs. 2.7%; P = 0.01). Conversely, there were lower associations of recreational drug use (10.0% vs. 17.0%; P = 0.01) and diabetes mellitus (DM) (35.9% vs. 45.9%; P = 0.01). Simple linear regression found that the SCa group had lower length of stay (LOS) (β = –0.08; P = 0.03). Logistic regression model showed that having cancer increased odds of all-cause mortality (odds ratio (OR) 1.82, 95% confidence interval (CI) 1.35–2.46; P < 0.01); however, the SCa group had comparable readmission rates, bloodstream infection, and in-hospital mortality.

**Conclusion:**

Compared to patients with sepsis without cancer, patients with sepsis and cancer have higher association with older age, male sex, White race, lower BMI, and TIA, and lower association with recreational drug use and DM. Patients with sepsis and cancer have lower LOS, higher all-cause mortality and have no difference in readmissions, bloodstream infections, and in-hospital mortality.

## Introduction

Sepsis is a dysregulated host response to infection resulting in organ dysfunction. It affects 50 million people and accounted for 20% of deaths worldwide prior to the COVID-19 pandemic [1]. Cancer and its various treatment modalities, including chemotherapy, radiation therapy, and immunotherapy, increase susceptibility to the development of sepsis. Historically, cancer patients are 10 times more likely to develop sepsis than the general population [2]. Furthermore, an epidemiological study found that of the 1.1 million sepsis-related hospitalizations in the United States, over 230,000 were admissions among cancer patients. This means that cancer patients comprise more than 20% of patients hospitalized for sepsis [3]. Of those cancer patients hospitalized with sepsis, 63.4% had solid tumors, 18.4% had hematologic malignancies, and the remainder were unknown [3]. Cancer patients are also more likely to experience adverse outcomes secondary to sepsis, specifically increased mortality rates. Compared to sepsis patients without cancer, sepsis patients with cancer have a significantly higher in-hospital mortality rate [3]. Some studies estimate that approximately 30% of deaths in cancer patients are due to sepsis [4]. Based on subtypes, hematologic mortality rate is higher than solid tumor mortality rate though this number has been declining over the years [5]. One prospective meta-analysis also showed significant increase in mortality rate in cancer patients, specifically a significant increase in late mortality but not early mortality [6]. Another study that examined cancer patients admitted to several European intensive care units (ICU) found that their 30-day mortality was as high as 40%. Use of mechanical ventilation and vasopressors in these patients was associated with increased risk for 30-day mortality. However, type of cancer, stem cell transplant status, and presence or absence of neutropenia did not significantly affect the 30-day mortality [7]. A meta-analysis of nine studies between 2015 and 2021 showed that the presence of comorbid lung or renal disease in cancer patients significantly increased mortality [6]. Delirium also leads to significant increase in mortality in critically ill patients with cancer [8]. Although the current literature clearly demonstrates an increased mortality rate among patients with sepsis who had cancer compared to those without cancer, there are limited data on specific risk factors or comorbidities that may contribute to the increased incidence of sepsis in cancer patients. Furthermore, studies focused on additional inpatient outcomes, such as in-hospital versus all-cause mortality, length of stay (LOS), readmission rates, and transition to palliative care are limited. Because of the significant and complex relationship between sepsis and cancer, we conducted this hypothesis generating study, and aimed to identify the risk factors and comorbidities among cancer patients with sepsis (SCa), as well as investigated multiple outcomes for sepsis, such as the rates of in-hospital mortality, all-cause mortality, hospital LOS, hospital readmission, palliative care, and bloodstream infection in cancer patients with sepsis compared to patients with sepsis who did not have cancer (SnoCa).

## Materials and Methods

### Study design and setting

Our study was a retrospective, cohort analytic study that utilized convenience sampling of the existing electronic medical records of an entire cohort of adult patients with sepsis who received care in an urban non-profit tertiary healthcare system.

### Participants

The inclusion criteria of our study were patients aged 18 years and older who had received in-hospital care for sepsis between January 1, 2023, and June 30, 2023. Sepsis was defined as per the Sepsis-3 consensus as life-threatening organ dysfunction caused by a dysregulated host response to infection in which the life-threatening acute organ dysfunction was identified by an increase in the total Sequential (Sepsis-related) Organ Failure Assessment Score (SOFA) of 2 points or more [9]. The exclusion criteria of our study were patients younger than 18 years of age and patients without a diagnosis of sepsis.

### Variables

Data collection included patient demographics, laboratory values, comorbid medical conditions, types of cancers in patients who had a history of cancer, types of treatment for cancer, and outcomes, such as in-hospital mortality, all-cause mortality, hospital LOS, hospital readmission, and bloodstream infection. We utilized Microsoft Excel (2016, Redmond, Washington, USA) spreadsheet to record all the data.

### Data source and access

This study was reviewed and approved by the Institutional Review Board (IRB) of our healthcare system, who granted authorization to collect data for research purposes only. Our study met international ethical guidelines and was performed in accordance with the Declaration of Helsinki. All investigators had full access to the data available only in the electronic medical records (Epic healthcare software, Epic Systems Corporation, Wisconsin, USA) of the list of patients approved by the medical informatics department, who were selected based on the inclusion criteria of the study. Being a retrospective data collection, informed consents were not required.

### Bias

In order to minimize potential confounders and ensure accurate identification of the diagnosis of sepsis, we carefully reviewed each medical record and excluded patients who presented with a wide range of noninfectious disorders which could mimic sepsis and were documented as “suspected sepsis” due to their presentation with similar clinical features such as fever, tachycardia, hypotension, and organ dysfunction. Such cases presented as acute heart failure and myocardial infarction with shock and organ dysfunction; or acute pulmonary embolism with hypoxemia and hypotension; or acute pancreatitis and gastrointestinal hemorrhage accompanied with systemic inflammatory response syndrome (SIRS) criteria and organ dysfunction; or acute thyroid storm, adrenal insufficiency, and diabetic ketoacidosis; or certain drug reactions and toxicities that presented with neuroleptic malignant syndrome, malignant hyperthermia, medication toxicity, and withdrawal syndromes; or acute episode of inflammatory vasculitis and autoimmune diseases that presented with fever, hypotension, and multiorgan involvement; or major trauma and hemorrhage which presented with shock and SIRS without infection.

### Study size

We selected the entire cohort of 1,001 adult patients with a diagnosis of sepsis who received care in our healthcare system within the study period. Upon further review, we found 938 patient records that had accurate and consistent documentation of sepsis and additional study variables. Hence, we included 938 patients.

### Statistical methods

We divided our cohort of patients into two groups: SCa and SnoCa. Statistical methods used for the study were independent *t*-test, Mann-Whitney U test, and Chi-square test. We used linear regression and logistic regression for modeling. The outcome analyzed using the linear regression was LOS, which was log-transformed due to a high level of skewness. The outcomes examined using the logistic regression were readmissions, bloodstream infections, and mortality (in-hospital and all-cause). The explanatory variables used were demographics, lifestyle habits, and morbidities. For the models, we consolidated different comorbidities into systemic groups, such as cardiovascular disorder group which included cerebrovascular accidents (CVAs), transient ischemic attack (TIA), peripheral arterial disease (PAD), hyperlipidemia, atrial fibrillation (Afib), coronary artery disease (CAD), heart failure with reduced ejection fraction (HFrEF), and heart failure with preserved ejection fraction (HFpEF); rheumatological disorder group which included systemic lupus erythematosus (SLE), rheumatoid arthritis (RA), mixed connective tissue disease (MCTD), Sjogren’s syndrome, vasculitis, and systemic sclerosis; respiratory disorder group which included chronic obstructive pulmonary disease (COPD), asthma, and obstructive sleep apnea (OSA); immunodeficiency disorder group which included human immunodeficiency virus infection (HIV), acquired immunodeficiency disorders, and common variable immunodeficiency disorder (CVID); and gastrointestinal disorder group which included gastroesophageal reflux disorder (GERD) and inflammatory bowel disease (IBD). We used SPSS 27 (Statistical Package for Social Sciences, version 27, IBM, Armonk, New York, USA) software for statistical analysis. The significance level was defined as P < 0.05.

## Results

A total of 938 patients were included in this study. There were 310 (33.0%) patients in the SCa group and 628 (67.0%) in the SnoCa group (Table 1). The mean age of the SCa group was significantly older compared to the SnoCa group (66.8 ± 13.6 vs. 61.5 ± 16.1 years; P < 0.01). There were more men in the SCa group compared to the SnoCa group (55.8% vs. 48.2%; P = 0.029). Most patients in both groups identified as White; however, there were more patients who identified as Black in the SCa group compared to the SnoCa group (29.0% vs. 27.9%; P < 0.01), while there were more patients who identified as Hispanic in the SnoCa group compared to the SCa group (15.2% vs. 6.8%; P < 0.01). There were no significant differences in the use of tobacco products and alcohol between the two groups. Use of recreational drugs was significantly higher in the SnoCa group compared to the SCa group (17.0% vs. 10.0%; P = 0.01). Average body mass index (BMI) was significantly higher in the SnoCa group compared to the SCa group (30.0 ± 10.4 vs. 28.1 ± 9.3 kg/m^2^; P = 0.01) (Table 1). Laboratory values, such as median white blood cell count, mean hemoglobin, median platelet count, median D-dimer, median prothrombin time, median activated partial thromboplastin time, and median lactate dehydrogenase showed no significant differences between the two groups (Table 1).

**Table 1:** Baseline Characteristics

Variable	Cancer (n = 310)	No cancer (n = 628)	P
Age (years), mean (SD)	66.78 (13.64)	61.47 (16.11)	< 0.01
Sex			0.03
Male, n (%)	173 (55.80)	303 (48.20)	
Female, n (%)	137 (44.20)	325 (51.80)	
Race			
White, n (%)	188 (60.60)	324 (51.70)	0.01
Black, n (%)	90 (29)	175 (27.90)	
Hispanic, n (%)	21 (6.80)	95 (15.20)	
Other, n (%)	11 (3.50)	33 (5.30)	
Social			
Tobacco use, n (%)	183 (59.20)	330 (52.60)	0.06
Alcohol use, n (%)	96 (31.10)	211 (33.60)	0.44
Recreational drug use, n (%)	31 (10.00)	107 (17.00)	0.01
BMI, mean (SD)	28.07 (9.27)	30.02 (10.38)	0.01
Lab values			
WBC (cells/mL) (median, 25th–75th)	15.3 (11.9–22.6)	15.9 (12.3–21.9)	0.09
Hb (g/dL), mean (SD)	10.1 (3.7)	9.9 (3.9)	0.31
Platelets (× 10^3^/µL) (median, 25th–75th)	108 (68–126)	103 (56–112)	0.06
D-dimer (µg (FEU)/mL) (median, 25th–75th)	4.4 (3.3–6.4)	4.1 (2.9–7.2)	0.13
PT (s) (median, 25th–75th)	14.2 (12.7–16.1)	13.9 (10.2–16.8)	0.23
aPTT (s) (median, 25th–75th)	38.4 (29.9–44.3)	37.2 (25.2–48.1)	0.94
LD (U/L) (median, 25th–75th)	691 (352–985)	728 (289–967)	0.39
Comorbidities			
Diabetes mellitus, n (%)	111 (35.9)	288 (45.9)	0.01
Hyperlipidemia, n (%)	171 (55.20)	333 (53.00)	0.54
HFrEF, n (%)	45 (14.50)	101 (16.10)	0.53
HFpEF, n (%)	40 (12.90)	81 (12.90)	0.99
CAD, n (%)	87 (28.10)	167 (26.60)	0.63
CVA, n (%)	50 (16.20)	126 (20.10)	0.15
TIA, n (%)	19 (6.10)	17 (2.70)	0.01
COPD, n (%)	83 (26.80)	163 (26.00)	0.79
Asthma, n (%)	43 (13.90)	115 (18.30)	0.09
OSA, n (%)	38 (12.30)	95 (15.10)	0.236
Hypothyroidism, n (%)	43 (13.90)	117 (18.70)	0.07
PAD, n (%)	32 (10.30)	92 (14.60)	0.07
Afib, n (%)	99 (31.90)	167 (26.60)	0.09
SLE, n (%)	4 (1.30)	12 (1.90)	0.49
RA, n (%)	16 (5.20)	21 (3.30)	0.18
MCTD, n (%)	0 (0.00)	1 (0.20)	1.00
Sjogren’s syndrome, n (%)	0 (0.00)	3 (0.50)	0.56
Vasculitis, n (%)	2 (0.60)	5 (0.80)	1.00
Systemic sclerosis, n (%)	1 (0.30)	2 (0.30)	1.00
CKD, n (%)	102 (32.90)	214 (34.10)	0.72
CLD, n (%)	52 (16.80)	128 (20.40)	0.19
Immunodeficiency, n (%)	17 (5.50)	28 (4.50)	0.49
HIV, n (%)	9 (2.90)	16 (2.50)	0.75
Chronic steroid use, n (%)	26 (8.40)	39 (6.20)	0.22
GERD, n (%)	105 (33.90)	220 (35.00)	0.73
IBD, n (%)	5 (1.60)	17 (2.70)	0.29

SD: standard deviation; BMI: body mass index; HFrEF: heart failure with reduced ejection fraction; WBC: white blood cell count; Hb: hemoglobin; PT: prothrombin time; aPTT: activated partial thromboplastin time; LD: lactate dehydrogenase; HFpEF: heart failure with preserved ejection fraction; CAD: coronary artery disease; CVA: cerebrovascular accident; TIA: transient ischemic attack; COPD: chronic obstructive pulmonary disease; OSA: obstructive sleep apnea; PAD: peripheral arterial disease; Afib: atrial fibrillation; SLE: systemic lupus erythematosus; RA: rheumatoid arthritis; MCTD: mixed connective tissue disease; CKD: chronic kidney disease; CLD: chronic liver disease; HIV: human immunodeficiency virus; GERD: gastroesophageal reflux disorder; IBD: inflammatory bowel disease.

We found a significantly higher frequency of TIA in the SCa group compared to the SnoCa group (6.1% vs. 2.7%; P = 0.01), and a higher frequency of diabetes mellitus (DM) in the SnoCa group compared to the SCa group (45.9% vs. 35.9%; P = 0.01). For the remaining comorbidities, there were no significant differences between the two groups (Table 1). In the SCa group, the three cancers with the highest frequencies were lung cancer (16.8%), colorectal cancer (11.6%), and breast cancer (9.4%) (Table 2). In the SCa group, 49.8% were previously treated with chemotherapy, 30% with radiotherapy, and 5.5% with hormonal therapy (Table 3).

**Table 2:** Types of Cancers in Patients With Cancer and Sepsis

Type of cancer	Frequency (n)	Percent (%)
Lung cancer	52	16.8
Colorectal cancer	36	11.6
Breast cancer	29	9.4
Prostate cancer	27	8.7
Skin cancer other than melanoma	25	8.1
Leukemias	21	6.8
Lymphomas	20	6.5
Gastroesophageal cancer	20	6.5
Urinary/bladder cancer	14	4.5
Uterine cancer	12	3.9
Kidney cancer	12	3.9
Esophageal cancer	12	3.9
Larynx cancer	10	3.2
Melanoma	10	3.2
Myelodysplastic syndrome	9	2.9
Liver cancer	9	2.9
Thyroid cancer	7	2.3
Brain cancer	7	2.3
Connective/soft tissue cancer	6	1.9
Anal cancer	4	1.3
Pharynx cancer	4	1.3
Multiple myeloma	4	1.3
Meningioma	3	1
Parotid gland cancer	2	0.6
Tonsil cancer	2	0.6
Tongue cancer	1	0.3

**Table 3:** Cancer Treatment Modality in Patients With Cancer and Sepsis

Type of treatment	Frequency (n)	Percent (%)
Chemotherapy	153	49.8
Radiotherapy	92	30
Hormonal therapy	17	5.5

A significantly greater percentage of patients in the SCa group were assigned to and received palliative care compared to SnoCa group (49.4% vs. 32.3%, P < 0.01) (Table 4). All-cause mortality was greater in the SCa group compared to the SnoCa group (66.1% vs. 53.2%, P < 0.01) (Table 4). Simple linear regression analysis showed that the SCa group had significantly lower hospital LOS (β = –0.08; P = 0.03) (Table 5). Logistic regression model showed that age, gender, race, BMI, alcohol or tobacco use, cardiovascular disorder, rheumatological disorder, respiratory disorder, immunodeficiency disorder, chronic kidney disease, and gastrointestinal disorder did not significantly affect LOS. Patients of older age had lower odds of readmission (odds ratio (OR) 0.98, 95% confidence interval (CI) 0.97–0.99; P < 0.01). Other comorbidities with greater odds of readmission included tobacco use (OR 1.42, 95% CI 1.04–1.93; P = 0.03), cardiovascular disorder (OR 1.83, 95% CI 1.24–2.70; P = 0.01), gastrointestinal disorder (OR 2.16, 95% CI 1.58–2.96; P < 0.01), and chronic kidney disease (OR 1.69, 95% CI 1.24–2.32; P = 0.01) (Table 6). Patients with elevated BMI had increased odds of bloodstream infection (OR 1.02, 95% CI 1.00–1.03; P = 0.02) (Table 7). Compared to patients who identified as White, patients who identified as Black had increased odds of in-hospital mortality (OR 1.90, 95% CI 1.38–2.62; P < 0.01) (Table 8) and all-cause mortality (OR 2.01, 95% CI 1.45–2.79; P < 0.01) (Table 9). Patients with chronic liver disease also had increased odds of in-hospital mortality as well (OR 1.58, 95% CI 1.10–2.26; P = 0.01) (Table 8). Patients with cancer had increased odds of all-cause mortality (OR 1.82, 95% CI 1.35–2.46; P < 0.01), as did patients with a respiratory disorder (OR 1.41, 95% CI 1.03–1.92; P = 0.03) (Table 9).

**Table 4:** Outcomes of Sepsis Patients With or Without Cancer

Outcome	SCa, n (%)	SnoCa, n (%)	P^a^
Palliative care	153 (49.40)	203 (32.30)	< 0.01
Readmission	108 (34.80)	223 (35.50)	0.84
Bloodstream infection	96 (31.00)	219 (34.60)	0.23
In hospital mortality	163 (52.60)	300 (47.80)	0.17
All-cause mortality	205 (66.10)	334 (53.20)	< 0.01

^a^Chi-square test.

**Table 5:** Length of Stay in Patients With Cancer and Sepsis

Risk factor	β	T-statistic	P
Age	–0.01	–0.32	0.75
Male sex	0.06	1.73	0.08
White vs. Black	0.03	0.96	0.34
White vs. Hispanic	–0.04	–1.24	0.21
White vs. other races	0.03	0.78	0.44
Body mass index	0.04	1.16	0.25
Alcohol use	0.05	1.51	0.13
Tobacco use	–0.02	–0.56	0.57
Cardiovascular disorder	0.03	0.81	0.42
Rheumatological disorder	–0.05	–1.36	0.17
Respiratory disorder	0.04	0.97	0.33
Immunodeficiency disorder	0.03	0.73	0.47
Chronic kidney disease	0.02	0.58	0.56
Chronic liver disease	–0.08	–2.25	0.03
Gastrointestinal disorder	0.04	0.97	0.33
Cancer	–0.08	–2.21	0.03

**Table 6:** Influence of Risk Factors on Readmissions

Risk factor	B	P	Exp(B)	95% CI for Exp(B)	
				Lower	Upper
Age	–0.02	< 0.01	0.98	0.97	0.99
Sex male	–0.19	0.22	0.83	0.62	1.12
White vs. Black	–0.24	0.17	0.78	0.56	1.11
White vs. Hispanic	0.022	0.92	1.02	0.65	1.60
White vs. other races	–0.72	0.07	0.49	0.23	1.06
Body mass index	< 0.01	0.97	1.00	0.99	1.02
Alcohol use	–0.27	0.10	0.77	0.56	1.05
Tobacco use	0.35	0.03	1.42	1.04	1.93
Cardiovascular disorder	0.60	0.01	1.83	1.24	2.70
Rheumatological disorder	0.28	0.34	1.32	0.74	2.36
Respiratory disorder	0.12	0.46	1.13	0.82	1.56
Immunodeficiency disorder	–0.43	0.19	0.65	0.34	1.26
Gastrointestinal disorder	0.77	< 0.01	2.16	1.58	2.96
Chronic kidney disease	0.53	0.01	1.69	1.24	2.32
Chronic liver disease	0.30	0.12	1.35	0.93	1.96
Cancer	0.06	0.72	1.06	0.77	1.45

CI: confidence interval.

**Table 7:** Influence of Risk Factors on Bloodstream Infection

Risk factor	B	P	Exp(B)	95% CI for Exp(B)	
				Lower	Upper
Age	–0.01	0.35	0.99	0.99	1.01
Sex male	–0.09	0.55	0.92	0.69	1.22
White vs. Black	–0.08	0.63	0.92	0.66	1.29
White vs. Hispanic	0.34	0.13	1.40	0.91	2.16
White vs. other races	0.58	0.08	1.78	0.94	3.39
Body mass index	0.02	0.02	1.02	1.00	1.03
Alcohol use	–0.13	0.43	0.88	0.65	1.20
Tobacco use	–0.13	0.39	0.88	0.65	1.18
Cardiovascular disorder	–0.03	0.87	0.97	0.66	1.39
Rheumatological disorder	–0.53	0.10	0.59	0.31	1.11
Respiratory disorder	–0.11	0.49	0.89	0.65	1.23
Immunodeficiency disorder	–0.41	0.21	0.66	0.34	1.27
Gastrointestinal disorder	–0.02	0.89	0.98	0.71	1.35
Chronic kidney disease	0.19	0.23	1.21	0.89	1.66
Chronic liver disease	0.27	0.15	1.31	0.91	1.88
Cancer	–0.09	0.56	0.91	0.67	1.24

CI: confidence interval.

**Table 8:** Influence of Risk Factors on In-Hospital Mortality

Risk factor	B	P	Exp(B)	95% CI for Exp(B)	
				Lower	Upper
Age	0.01	0.71	1.00	0.99	1.01
Sex male	0.21	0.14	1.23	0.94	1.63
White vs. Black	0.64	< 0.01	1.90	1.38	2.62
White vs. Hispanic	0.18	0.42	1.19	0.78	1.82
White vs. other races	0.42	0.20	1.52	0.80	2.90
Body mass index	0.01	0.33	1.01	0.99	1.02
Alcohol use	0.24	0.11	1.27	0.95	1.71
Tobacco use	0.05	0.76	1.05	0.79	1.39
Cardiovascular disorder	0.03	0.85	1.03	0.73	1.46
Rheumatological disorder	0.10	0.72	1.11	0.64	1.94
Respiratory disorder	0.02	0.88	1.02	0.76	1.39
Immunodeficiency disorder	0.19	0.53	1.21	0.67	2.19
Gastrointestinal disorder	–0.43	0.01	0.65	0.48	0.88
Chronic kidney disease	0.16	0.30	1.17	0.87	1.59
Chronic liver disease	0.45	0.01	1.58	1.10	2.26
Cancer	0.26	0.08	1.29	0.97	1.74

CI: confidence interval.

**Table 9:** Influence of Risk Factors on All-Cause Mortality

Risk factor	B	P	Exp(B)	95% CI for Exp(B)	
				Lower	Upper
Age	0.01	0.33	1.01	0.99	1.02
Sex male	0.23	0.12	1.25	0.95	1.66
White vs. Black	0.69	< 0.01	2.01	1.45	2.79
White vs. Hispanic	0.19	0.39	1.20	0.79	1.84
White vs. other races	0.24	0.48	1.27	0.66	2.43
Body mass index	0.01	0.65	1.00	0.99	1.02
Alcohol use	–0.02	0.92	0.99	0.73	1.33
Tobacco use	0.12	0.43	1.12	0.84	1.50
Cardiovascular disorder	–0.02	0.91	0.98	0.69	1.39
Rheumatological disorder	0.10	0.72	1.11	0.63	1.95
Respiratory disorder	0.34	0.03	1.41	1.03	1.92
Immunodeficiency disorder	–0.08	0.79	0.92	0.51	1.69
Gastrointestinal disorder	–0.40	0.01	0.67	0.49	0.91
Chronic kidney disease	0.15	0.35	1.16	0.85	1.57
Chronic liver disease	0.35	0.06	1.42	0.99	2.06
Cancer	0.60	< 0.01	1.82	1.35	2.46

CI: confidence interval.

## Discussion

In this study, we found that SCa patients were older compared to SnoCa patients. An epidemiological study reported that among patients hospitalized for sepsis, the mean age for patients with cancer was significantly higher than that of patients without cancer [5]. Another nationwide intensive care unit (ICU) cohort study also reported similar findings [10]. In both studies, the age differential was most highly pronounced in patients with solid tumors compared to those with hematologic malignancies. A retrospective cohort study correspondingly demonstrated that patients with solid tumors presented with sepsis at a significantly older age (age 67) than patients with hematologic malignancies (age 55) [11]. Our findings are consistent with the existing literature. Interestingly, we found that SCa patients of older age had lower odds of readmission. However, most literature had reported that SCa patients had higher odds of readmission, and that malignancy was an independent predictor of increased readmissions [12–15]. Our unique findings can be explained by increased rates of all-cause mortality and palliative care in the SCa group, which likely reduced the number of patients who were eligible for readmission. This finding is compatible with cohort studies that have shown that older patients with cancer and sepsis have increased in-hospital and short-term mortality and are more likely to transition to hospice services [10, 16]. Therefore, the decreased readmission rate in the SCa group does not reflect a lower rate of post-hospitalization complications, but rather decreased need for future readmissions.

We found that SCa patients were mostly men. In a study of patients with fever of unknown origin which was later identified to be due to malignancy, 56.5% of the patients were men [17]. In another study, out of all the cancer patients who developed sepsis, 53.3% of patients were men [18]. Another study reported that the incidence of sepsis within 1 year in cancer patients was greater in men [19]. Perhaps the prevalence of the types of cancers could explain why cancer patients with sepsis were most likely to be men. Based on these studies, association of sepsis in men with cancer could have been linked to the types of cancers, such as hematologic malignancies, which were more commonly seen in men [18]. While the National Comprehensive Cancer Network (NCCN) guidelines detail the increased risk of sepsis in patients with hematologic and solid malignancies, they do not comment on sex differences. However, the consistent finding across epidemiologic and clinical studies is that male sex is an independent risk factor for sepsis in cancer patients. The mechanisms underlying this disparity remain incompletely understood, but may involve biological, behavioral, and healthcare access factors.

The finding that SCa patients tended to be of White race contrasts with the previously reported literature, which reported a higher incidence of sepsis among non-White cancer patients [20–23]. However, epidemiologic studies utilizing the REGARDS cohort found that in community-dwelling cancer survivors, race had minimal to no impact in sepsis risk or incidence after cancer survival [20, 21]. Another study demonstrated that Native Americans diagnosed with cancer had a two-fold higher risk of sepsis compared with other ethnic groups, including Whites [22]. Additionally, one study reported that Black patients with cancer had the highest age adjusted risk of sepsis compared to American Indian or Alaskan Native, White, and Asian/Pacific Islander patients with cancer [23]. Several studies have also suggested how Black patients with cancer experience a higher incidence of sepsis and poorer outcomes due to delayed cancer treatment [20]. A possible explanation for the association in our study could be the fact that our healthcare system is affiliated with a multispecialty cancer care center, which falls within the geographic proximity of many cancer patients in our region, who happen to be mostly White individuals. Factors, such as access to care and regional population composition might have contributed to this finding in our study. More research is needed to further elucidate the impact of race on the association between various types of malignancies and sepsis.

Our study revealed that SCa patients had a lower BMI compared to the SnoCa group. Studies have reported that individuals with sepsis might have decreased BMI due to systemic inflammation promoting metabolic alterations that influence energy use and facilitate catabolism [24, 25]. However, individuals with cancer tend to have a decreased BMI due to cancer cachexia, which is a result of pro-inflammatory mechanisms, an alteration in hypothalamic appetite control, and symptoms of nausea, dysgeusia, and depression [26, 27]. Various studies have investigated the role of BMI in the context of sepsis and cancer individually and concurrently. One study showed that increased BMI was associated with an increased risk of sepsis [28]. Another study revealed that excess body fat is linked to increased risk of various cancers [29]. These results have clinical implications. While an increased BMI increases an individual’s risk of both sepsis and cancer individually, patients presenting with either sepsis or cancer who have an elevated BMI may counterintuitively benefit from their elevated BMI. A phenomenon, known as the “obesity paradox,” can be observed in both sepsis and cancer. The “obesity paradox” is an observation that patients with an elevated BMI have a protective effect compared to individuals with normal or lower BMIs [30]. Studies have reported that septic patients with a higher BMI had a lower risk of death [31, 32]. Proposed mechanisms suggest that elevated BMI modifies immune function by decreasing the risk of excessive inflammation [33]. Additionally, elevated adipose tissue might be beneficial in the setting of the catabolic strain caused by sepsis [34]. One meta-analysis reported that while high BMI was associated with increased mortality in specific cancers, obese patients with renal cell carcinoma, lung cancer, and melanoma interestingly had higher survival rates [35]. Underlying mechanisms thought to facilitate this include greater tolerance towards immunotherapy [36, 37]. Remarkably, amongst patients with both sepsis and cancer, elevated BMI has been reported to be associated with improved survival [30]. Thus, our findings align with the relationship between BMI, sepsis, and cancer.

In our study, patients with elevated BMI had increased odds of bloodstream infection. This finding has not been reported. Though obese and underweight people may be at higher risk of infections compared to those of normal BMI, some studies have reported that people with obesity were more likely to contract intra-abdominal infections and skin and soft tissue infections [38]. This is believed to be due to alterations in immune response and inflammation [38]. The innate and adaptive immune responses and cytokine production are affected by obesity [39]. Those who are overweight have higher risk of having diabetes and cardiovascular disease, which are known to influence the risk of infection, healing, and immunity [39]. Elevated BMI is associated with many comorbidities that are ultimately thought to alter pathophysiology in a way that increases risk of bloodstream infection [39].

The observed association between TIA and SCa patients appears to be a novel finding. While cancer is a well-established risk factor for ischemic stroke, studies directly linking sepsis to TIA are limited. Cancer creates a prothrombotic state through tumor-derived procoagulant mediators, systemic inflammation, and endothelial activation, predisposing to arterial and venous thromboembolism [40–44]. Biomarkers such as D-dimer, lactate dehydrogenase, and inflammatory cytokines are frequently elevated in cancer-related cerebrovascular events, supporting the role of hypercoagulability and microembolism in TIA and stroke [43–46]. Sepsis may compound these risks by driving endothelial injury, immune dysregulation, and coagulopathy, thereby creating conditions favorable for cerebral ischemia [43, 44, 47]. Endothelial dysfunction, reflected by increased soluble ICAM 1, VCAM 1, and thrombomodulin, is observed in cancer-related TIA and may be further exacerbated by sepsis [44–46]. Tumor derived extracellular vesicles have been implicated in cancer associated thrombosis through tissue factor independent pathways, and their levels correlate with D-dimer concentrations and stroke risk [48]. Likewise, conditions such as nonbacterial thrombotic endocarditis and paradoxical embolism, which are more prevalent in the setting of systemic inflammation, may provide alternative embolic sources in this patient group [48–50]. Taken together, the interplay of cancer associated with hypercoagulability, sepsis driven immune-thrombosis, and endothelial dysfunction provides a plausible explanation for the observed link. Having a history of TIA may indicate a broader bidirectional relationship between cerebrovascular disease and sepsis in cancer patients, as well as its association with worse outcomes.

In SCa patients, comorbidities with greater odds of readmission included tobacco usage, cardiovascular disorder, gastrointestinal disorder, and chronic kidney disease. Studies indicate that both former and current tobacco use has been associated with increased odds of 30-day unplanned hospital readmissions in SCa patients compared to never smokers, even after adjusting for covariates [51]. This is likely due to the immunosuppressive and pro-inflammatory effects of smoking, which leads to recurrent infections or delayed recovery in the setting of sepsis. Implementation of inpatient smoking cessation services has been shown to reduce 30-day readmission rates in tobacco users, including those with cancer [52].

Cardiovascular disease, especially coronary artery disease and heart failure, is a well-established risk factor for hospital readmission in sepsis survivors both with and without cancer [13, 53]. However, the risk is still greater in patients who also have cancer compared to those who do not, with malignancy and cardiovascular disease both independently increasing risk [13]. Although the odds between the two groups have not been consistently reported in the literature, the consensus suggests that there is a synergistic effect between cancer and cardiovascular disease [14]. Cancer likely increases this risk in patients with cardiovascular disease due to cancer-related immune dysregulation, the pro-inflammatory state of malignancy, and the cardiotoxic side effects of chemotherapies.

Gastrointestinal disorder, especially cirrhosis and liver disease, is associated with increased odds of readmission in sepsis survivors. Current literature shows that this risk is elevated in patients with cancer, though there are no data that directly compare the SCa and SnoCa groups [10, 13]. The increased rate of readmission in cancer patients with concomitant gastrointestinal disorder can potentially be due to increased abdominal infectious sources of sepsis in this group, as well as treatment-related gastrointestinal and hepatic adverse effects [2].

Chronic kidney disease is also associated with an increased odds of hospital readmission in SCa patients compared to SnoCa patients [13, 14, 53]. A comparative study has reported that chronic kidney disease increases risk of recurrent infection and hospitalizations, and that this risk is elevated for cancer patients compared to non-cancer patients [54]. This is likely due to cancer-mediated immune dysregulation in the setting of an immunosuppressed state both from underlying chronic kidney disease, cancer treatment, and the cancer itself.

We found that SCa patients had shorter hospital LOS than SnoCa patients. This is contrary to most literature which reported that SCa patients generally have similar or slightly longer LOS than SnoCa patients [10, 16, 55]. This is especially true for patients with solid tumors. However, one study in the ICU setting found that patients with metastatic disease had shorter LOS [55]. Although not all the patients in our study had metastatic disease, a potential explanation for our unique finding is that more advanced disease led to treatment limitations or quicker transition to hospice. It is also possible that SCa patients received quicker escalation of care, leading to more prompt source control and a shorter hospital LOS.

We also found that, compared to patients who identified as of White race, patients who identified as Black race had increased odds of in-hospital mortality and all-cause mortality. Reports from prior studies assessing mortality differences between different races have been inconsistent [56]. This could be due to variable methods of classifying patients as sepsis versus no sepsis in these studies. In some cases, there were no significant differences in mortality between Black patients and White patients once factors such as poverty and insurance access were accounted for. There were several studies that showed higher incidence of sepsis and hospitalization rates in patients who identified as non-White compared to those who identified as White [56]. In this large population study, Black patients had higher 90-day mortality compared to White patients. The study suggested that delays in recognizing sepsis in Black patients leading to delays in antibiotic administration was a major driver in the higher 90-day mortality [56]. More studies are needed to investigate racial disparities in patients admitted with sepsis with or without cancer.

Our data showed that SCa patients had increased odds of all-cause mortality. This group of patients tend to be more susceptible to infections due to being immunosuppressed from chemotherapy. Cancer itself may lead to a weakened immune system. Systematic reviews and meta-analysis have shown variability in mortality rates in SCa patients compared to SnoCa patients [12]. This could be due to variable methods of defining sepsis in the inpatient setting. Some hospitals used systemic inflammatory response syndrome (SIRS) criteria, while other hospitals relied on quick Sequential Organ Failure Assessment (qSOFA) criteria [57]. A uniform standardized and structured strategy is needed to identify patients with sepsis, especially those with cancer who may be at higher risk of contracting infections [57, 58].

Being a retrospective study, our study had some limitations. We had to rely upon the entries in the medical records made by the care team that provided care in an urban tertiary care setting, hence our findings cannot be generalized. A large sample size that received care at one institution strengthened the findings of our study, along with proper documentation of all the events preceding, during, and following the sepsis admission in both the groups, which strengthened our study and allowed us to capture the appropriate data variables.

### Conclusion

We conclude that compared to patients with sepsis without cancer, patients with sepsis and cancer have higher association with older age, male sex, White race, lower BMI, and TIA, and lower association with recreational drug use and DM. Patients with sepsis and cancer have lower LOS, higher all-cause mortality and have no difference in readmissions, bloodstream infections, and in-hospital mortality. Greater index of suspicion and early management of patients with sepsis and cancer with nonmodifiable risk factors and optimization of modifiable risk factors may offer favorable outcomes. Further multicenter and hypothesis-driven studies are needed in diverse population settings to investigate these risk factors and outcomes, especially, the novel findings of higher prevalence of White race and history of TIA in patients with sepsis and cancer.

## Data Availability

The authors declare that data supporting the findings of this study are available within the article.
